# Macroalgal protein hydrolysates from *Palmaria palmata* influence the ‘incretin effect’ in vitro via DPP-4 inhibition and upregulation of insulin, GLP-1 and GIP secretion

**DOI:** 10.1007/s00394-021-02583-3

**Published:** 2021-06-03

**Authors:** C. M. McLaughlin, P. A. Harnedy-Rothwell, R. A. Lafferty, S. Sharkey, V. Parthsarathy, P. J. Allsopp, E. M. McSorley, R. J. FitzGerald, F. P. M. O’Harte

**Affiliations:** 1 School of Biomedical Sciences, Ulster University, Cromore Road, Coleraine, Co. Derry, BT52 1SA Northern Ireland; 2 Department of Biological Sciences, University of Limerick, Castletroy, Limerick Ireland; 3 Health Research Institute (HRI), University of Limerick, Limerick, Ireland; 4 Nutrition Innovation Centre for Food and Health, School of Biomedical Sciences, Ulster University, Cromore Road, Coleraine, Co. Derry, BT52 1SA Northern Ireland

**Keywords:** Antidiabetic, Dipeptidylpeptidase-4, Dulse, Incretin secretion, *Palmaria palmata*, Protein hydrolysate, Type 2 diabetes

## Abstract

**Purpose:**

This study investigated metabolic benefits of protein hydrolysates from the macroalgae *Palmaria palmata*, previously shown to inhibit dipeptidylpeptidase-4 (DPP-4) activity in vitro.

**Methods:**

Previously, Alcalase/Flavourzyme-produced *P. palmata* protein hydrolysate (PPPH) improved glycaemia and insulin production in streptozotocin-induced diabetic mice. Here the PPPH, was compared to alternative Alcalase, bromelain and Promod-derived hydrolysates and an unhydrolysed control. All PPPH’s underwent simulated gastrointestinal digestion (SGID) to establish oral bioavailability. PPPH’s and their SGID counterparts were tested in pancreatic, clonal BRIN-BD11 cells to assess their insulinotropic effect and associated intracellular mechanisms. PPPH actions on the incretin effect were assessed via measurement of DPP-4 activity, coupled with GLP-1 and GIP release from GLUTag and STC-1 cells, respectively. Acute in vivo effects of Alcalase/Flavourzyme PPPH administration on glucose tolerance and satiety were assessed in overnight-fasted mice.

**Results:**

PPPH’s (0.02–2.5 mg/ml) elicited varying insulinotropic effects (*p* < 0.05–0.001). SGID of the unhydrolysed protein control, bromelain and Promod PPPH’s retained, or improved, bioactivity regarding insulin secretion, DPP-4 inhibition and GIP release. Insulinotropic effects were retained for all SGID-hydrolysates at higher PPPH concentrations. DPP-4 inhibitory effects were confirmed for all PPPH’s and SGID counterparts (*p* < 0.05–0.001). PPPH’s were shown to directly influence the incretin effect via upregulated GLP-1 and GIP (*p* < 0.01–0.001) secretion in vitro, largely retained after SGID. Alcalase/Flavourzyme PPPH produced the greatest elevation in cAMP (*p* < 0.001, 1.7-fold), which was fully retained post-SGID. This hydrolysate elicited elevations in intracellular calcium (*p* < 0.01) and membrane potential (*p* < 0.001). In acute in vivo settings, Alcalase/Flavourzyme PPPH improved glucose tolerance (*p* < 0.01–0.001) and satiety (*p* < 0.05–0.001).

**Conclusion:**

Bioavailable PPPH peptides may be useful for the management of T2DM and obesity.

**Supplementary Information:**

The online version contains supplementary material available at 10.1007/s00394-021-02583-3.

## Introduction

Type 2 diabetes mellitus (T2DM) is a metabolic disorder of complex aetiology characterised by a deficiency, and/or dysfunction of endogenous insulin and glucagon production [1]. In diabetes, loss of insulin-producing pancreatic beta-cell mass [2], is accompanied by dysfunction of glucagon-producing alpha cells, which fail to respond to the normal suppressive effects of glucose and insulin, leading to hyperglucagonaemia [3]. Best-estimates state that there are around 463 million adults globally living with diabetes, projected to rise to 700 million by 2045 [4], with T2DM representing ~ 90% of cases. The economic burden of diabetes on global healthcare systems is considerable, with a minimum of $760 billion USD attributed to spending on the disease in 2019, equating to 10% of global adult healthcare costs [4]. An important factor in this spending arises from costs amassed from treatment of microvascular (retinopathy, nephropathy, and neuropathy) and macrovascular (coronary artery disease, stroke, and peripheral vascular disease) complications [5]. Preventative strategies, coupled with earlier diagnosis and novel treatments, have the potential to reduce the occurrence of these complications [6].

Lifestyle interventions such as increased physical activity and improved, nutritionally balanced diets are considered first-line options in the prevention and treatment of T2DM [7, 8]. While high-quality dietary protein is an integral part of any such diet [9], it has also been established that a high-protein diet can lower postprandial blood glucose in T2DM and improve overall glucose and lipid metabolism [10, 11]. Beyond dietary protein, protein hydrolysates, peptides and single amino acids can beneficially regulate glycaemia, with the magnitude of response differing significantly depending on the primary sequence of peptides and specific amino acids generated following digestion [12, 13].

Mechanisms determining glycaemic improvements of various protein hydrolysates have been established, highlighting the importance of inhibitory actions on the ubiquitous enzyme dipeptidylpeptidase-4 (DPP-4) [14–18]. DPP-4 inhibition has become a staple of diabetes management, with a plethora of drugs now available since the approval of sitagliptin (Januvia®) in 2006 [19]. Success of DPP-4 inhibition lies in the preservation of the “incretin effect”, which promotes a rise in plasma insulin following food intake [20]. The rise in plasma insulin not only reflects a response to increased postprandial glucose, but approximately 50% of the overall insulinotropic response is attributed to the release of two gut-derived hormones, namely: glucagon-like peptide 1 (GLP-1) and glucose-dependent insulinotropic polypeptide (GIP) [20]. Both GLP-1 and GIP are inactivated following N-terminal dipeptide removal by DPP-4 [19]. Furthermore, DPP-4 resistant GLP-1 receptor agonists (incretin mimetics) have also been developed and are widely prescribed for T2DM management [21–23].

It has recently been uncovered that, beyond DPP-4 inhibition, protein hydrolysates from underutilised marine sources, such as blue whiting, boarfish and salmon skin, can directly influence glycaemia through improved insulin production and secretion coupled with upregulated GLP-1 secretion in both in vitro [24–26] and in vivo settings [25, 27]. The present study has sought to establish whether crude hydrolysates of the macroalgae *Palmaria palmata* can replicate the effects of piscine-derived protein hydrolysates.

*P. palmata* (dulse) has become popular as a foodstuff due to its relatively high protein content [28], in addition to being a potential source of biofunctional proteinaceous and antioxidant ingredients [29–31]. Notably, both crude hydrolysates of *P. palmata* [32] and isolated peptides from this source have demonstrated an ability to inhibit DPP-4 in vitro [33]. Furthermore, twice daily, chronic administration of a crude *P. palmata* protein hydrolysate, Alcalase/Flavourzyme PPPH, has been shown to improve glycaemic control in streptozotocin-induced diabetic mice [34]. Thus, the present study aims to employ a number of established screening methods to uncover the specific mechanisms responsible for the positive glycaemic effects of PPPH’s and identify the hydrolysate which shows greatest anti-diabetic potential.

## Materials and methods

### Materials and chemicals

H-Gly-Pro-AMC (7-amino-4–methyl coumarin) and Diprotin A were obtained from Bachem Feinchemikalien (Bubendorf, Switzerland). Promod 144 MG provided by Biocatalysts Ltd. (Cardiff, Wales, UK). HPLC grade water and acetonitrile from VWR International (Dublin, Ireland) and trinitrobenzenesulphonic acid (TNBS) reagent was from Medical Supply Co Ltd. (Dublin, Ireland). Calcium chloride dihydrate (CaCl_2_ × 2H_2_O), d-glucose, HEPES, hydrochloric acid (HCl), magnesium sulphate (MgSO_4_ × 7H_2_O), potassium dihydrogen orthophosphate (KH_2_PO_4_), potassium chloride (KCl), sodium bicarbonate (NaHCO_3_) and sodium chloride (NaCl) were purchased from BDH Chemicals Ltd. (Poole, Dorset, UK). Foetal bovine serum (FBS), Hank's buffered saline solution (HBSS 10X stock), penicillin–streptomycin (0.1 g/l), RPMI-1640 culture media, Dulbecco's modified Eagle's medium (DMEM)-containing high glucose and trypsin/EDTA (10X) were obtained from Gibco Life Technologies Ltd. (Paisley, Strathclyde, UK). Radio-labelled sodium iodide (Na^125^I, IMS 100 mCi/ml stock) was from Perkin Elmer (Buckinghamshire, UK). Rat insulin standard was from Novo Industria, Copenhagen, Denmark. All other reagents including DPP-4, from porcine kidney (≥ 10 units/mg protein), Alcalase® 2.4 L and Flavourzyme® 500 L supplied by Sigma Chemical Company Ltd. (Wicklow, Ireland). Air-dried milled (5 mm) *P. palmata* sample was purchased from Irish Seaweeds Ltd., Belfast, Co. Antrim, N. Ireland. The macroalgae was further milled with a Cyclotec™ Mill (1 mm screen, FOSS Tecator AB, Hoganas, Sweden) and stored at RT.

### Preparation of crude aqueous soluble protein extracts

Crude aqueous and alkaline soluble protein extracts were prepared using the method described previously [32]. Milled *P. palmata* powder was suspended at a mass:volume ratio of 1:20 ((w/v), 1 kg:20.0 l) and gently agitated at room temperature for 3 h. The supernatant containing the aqueous soluble protein was acquired following centrifugation at 4,190 × g (Sorvall RC6 Plus, Fisher Scientific, Dublin, Ireland) for 15 min at RT. The pellet was resuspended in 0.12 M NaOH (1:15 (w/v)) and gently agitated for 1 h at RT and supernatant containing the alkaline soluble protein was acquired following centrifugation. The pellet was subjected to a second alkaline extraction, and both supernatants combined. A double isoelectric precipitation step was utilised to semi-purified and concentrated aqueous (pH 2.5) and alkaline (pH 4.0) soluble protein components. The precipitated protein pellets obtained following the second isoelectric precipitation were resuspended in dH_2_O to a protein concentration of ~ 2.4% (w/v) and combined. Protein concentration was determined by the modified Lowry protein quantification method as described previously [35]. Samples were analysed in triplicate.

### Enzymatic hydrolysis of macroalgal proteins and simulated gastrointestinal digestion

Macroalgal protein was hydrolysed as described previously [32]. A 2% (w/v) protein solution was preheated to 50°C and adjusted to pH 7.0 and hydrolysed with Alcalase 2.4 L, Alcalase 2.4 L and Flavourzyme 500 L, bromelain and Promod 144 MG at an enzyme:substrate (E:S) ratio of 1:100 (w/w or v/w) for 4 h at 50°C. The reaction was maintained at pH 7.0 using a pH–stat (842 Titrando, Metrohm, Switzerland) and enzyme inactivated by heating at 90°C for 20 min. A control protein sample, containing no proteolytic enzyme, was treated in the same manner. All samples were subsequently freeze–dried (FreeZone 18L, Labconco, MO, USA) and stored at − 20 °C.

To assess oral bioavailability, PPPH’s were subjected to simulated gastrointestinal digestion (SGID), described previously [36]. In brief, unhydrolysed protein controls and hydrolysates were diluted to 2.0% (w/v) protein in water and incubated at 37°C and pH 2 for 90 min with pepsin at an E:S of 1:40 (w/w). The samples were adjusted to pH 7 and subjected to heat inactivation at 90°C for 20 min. The samples were incubated for a further 150 min at 37 °C with Corolase PP (E:S of 1% (w/w)). SGID samples were heat inactivated and all samples were subsequently freeze-dried and stored at − 20°C.

### Physicochemical characterisation of PPPH

The molecular mass distribution profile of the hydrolysates and their SGID samples were determined by gel permeation–high-performance liquid chromatography (GP-HPLC) as described previously [37]. The amino nitrogen content of PPPH was estimated by the TNBS method with absorbance readings taken at 350 nm [38]. Samples were analysed in triplicate.

### Insulin secretion studies in clonal pancreatic cells

Insulinotropic effects of PPPH and SGID samples were measured in vitro using clonal pancreatic BRIN-BD11 cells [39]. BRIN-BD11 cells (1.5 × 10^5^ cells/well) were incubated for 20 min with a range of PPPH concentrations (0.039–2.5 mg/ml) in the presence of 5.6 mM glucose at 37°C. Following incubation, supernatant (900 µL) was withdrawn and frozen at − 20°C until required. Insulin was quantified using a dextran-coated charcoal radioimmunoassay (RIA), using crystalline rat insulin standard, guinea-pig anti-porcine antiserum (1:30,000 dilution) and ^125^I-bovine standard (10,000 cpm), described previously [40]. The concentration of insulin in each sample was determined in duplicate from the prepared insulin standard curve ranging from 0.039–20 ng/ml.

### Cellular toxicity via MTT assay

To determine cytotoxicity of PPPH and SGID samples on BRIN-BD11 cells, the MTT (3-(4,5-Dimethylthiazol-2-yl)-2,5-Diphenyltetrazolium Bromide) assay was employed. A fixed dose of PPPH was prepared in Krebs Ringer bicarbonate buffer (KRBB) buffer supplemented with 5.6 or 16.7 mM glucose. Upon completion of co-incubation, KRBB was removed and cells washed with HBSS. Growth media (100 μl) was added to each well of a 96 well plate and further supplemented with 20 μl of MTT solution (5 mg/ml stock). Plates were incubated for 2 h in a modified atmosphere (95% O_2_, 5% CO_2_) tissue culture incubator at 37°C. MTT/growth media was aspirated and washed for a final time with HBSS. The formazan crystals developed were then dissolved using 100 μl of DMSO and the plate agitated at RT for 5 min. Plates were read on a spectrophotometer with absorbance set at 570 nm.

### Quantification of DPP-4 inhibition

DPP-4 inhibition was determined as described previously [33]. Activity was expressed as IC_50_ values for three independent replicates (n = 3). Diprotin A was used as a positive control.

### In vitro GLP-1 secretion from GLUTag cells and GIP secretion from STC-1 cells

In vitro effects of the PPPH and the SGID samples on GLP-1 secretion were measured using the murine enteroendocrine GLUTag cell line [41], kindly gifted by Prof. Fiona Gribble/Daniel Drucker. Cells were cultured in high glucose (25 mM) Dulbecco’s Modified Eagle’s Medium (without glutamine), as described previously [42]. Cells were seeded into 24-well plates (1.5 × 10^5^ cells/well) attaching over 36 h at 37°C. Following a pre-incubation step (1.1 mM glucose solution in KRBB for 40 min at 37°C), cells were incubated with the PPPH and SGID samples (2.5 mg/ml) prepared in 2 mM glucose followed by 2 h incubation at 37°C. Thereafter, 800 µl of supernatant was collected and subsequently used to measure total GLP-1 release by ELISA (Millipore, Hertfordshire, UK) as per manufacturer’s protocol.

STC-1 cells differentiate by secreting satiety and glucose homeostatic hormones such as CCK, GIP, PYY, GLP-1 and GLP-2 [43]. The experimental procedure was similar to the GLUTag screening procedure. After 2 h co-incubation, 800 μl of the supernatant was aspirated and stored at − 20°C before quantification using a GIP ELISA (Millipore, Hertfordshire, UK) as per manufacturer’s protocol.

### Glucose uptake study using differentiated adipocytes

Adipocyte (3T3-L1) cells were obtained from the American Type Culture Collection (ATCC, Manassas, Virginia, USA). 3T3-L1 cells were seeded in a 96, black-walled, clear bottom plates (2 × 10^4^ cell/well). Cells were maintained with Dulbecco’s Modified Eagle’s Medium (DMEM) supplemented with 10% (v/v) heat inactivated FBS. Cells were incubated for a further 2 days and then differentiated in DMEM containing 10% FBS, 15 μg/mL insulin, 1 μM dexamethasone and 0.5 mM 3-isobutyl-l-methylxanthine (IBMX). Cells were cultured in DMEM containing 10% FBS and 15 μg/mL of insulin. Cells were treated with the test sample (100 µl) or control which were supplemented in glucose-free culture medium containing 150 µg/ml fluorescently tagged 2-deoxyglucose analogue (2-NBDG) and incubated for 20 min. Plates were centrifuged for 5 min at 400 × G at RT. Supernatant was aspirated, and cells washed with 200 µl cell-based assay buffer followed by further centrifugation for 5 min. Wash buffer was removed and 100 µl of cell-based assay buffer was added to all wells and the fluorescence was read immediately at 485 nm with emission measured at 535 nm using the FlexStation scanning fluorimeter (Molecular Devices, Sunnyvale, CA, USA).

### Mechanistic studies using BRIN-BD11 cells

Effect of PPPH’s (2.5 mg/ml) on changes in membrane potential and intracellular calcium concentration [Ca^2+^] were determined fluorometrically utilising monolayers of BRIN-BD11 cells and Flex membrane potential and calcium assay kits (Molecular Devices, Sunnyvale, CA, USA), as previously described [44]. Assay choice was based on the knowledge that increased intracellular [Ca^2+^] is the primary insulin secretory signal, while cAMP signalling-dependent mechanisms are also critical for incretin-mediated insulin release [61]. Control cultures were 30 mM KCl and 10 mM alanine in the presence of 5.6 mM glucose. Fluorometric data were acquired using a FlexStation scanning fluorimeter utilizing an integrated fluid transfer workstation (Molecular Devices, Sunnyvale, CA, USA). The effect of the PPPH’s subjected to SGID on the production of cAMP was also assessed in BRIN-BD11 cells. Cells were seeded (1.5 × 10^5^ cells/well) into 24-well plates and incubated overnight. Cells were washed with HBSS before incubation (20 min, 37°C) with the PPPH (2.5 mg/ml) in the presence of 200 μM IBMX. Culture media was removed, cells lysed and the cAMP concentration in lysates was determined using a cAMP detection kit (R&D Systems Parameter, Abingdon, UK).

### Acute in vivo effects of a PPPH on glucose tolerance and satiety

NIH Swiss mice (Harlan UK Ltd., Blackthorne, UK) were employed for acute in vivo experiments. Animals (10–12 week old) were maintained in an environmentally controlled laboratory at 22 ± 2°C with a 12 h dark and light cycle with ad libitum access to standard rodent diet (10% fat, 30% protein and 60% carbohydrate: Trouw Nutrition, Northwich, UK) and drinking water. Acute glucose-lowering and insulin releasing properties of Alcalase/Flavourzyme PPPH was determined in age-matched groups (n = 8) of overnight-fasted mice, who received an oral gavage of either glucose alone (18.8 mmol/kg body weight) or in combination with PPPH (100 mg/kg bw). Blood glucose was measured using an Ascencia Contour blood glucose meter (Bayer Healthcare, Newbury, UK) and samples were collected via tail vein bleed in chilled fluoride/heparin micro-centrifuge tubes (Sarstedt, Numbrecht, Germany) and centrifuged at 13,000 rpm for 10 min. Plasma was aliquoted and stored at − 20 °C until required for insulin determination using a modified dextran-coated charcoal RIA [40].

Satiating effect of Alcalase/Flavourzyme PPPH was assessed in male HsD:Ola TO mice (10–12 weeks, Envigo, Blackthorn, UK), maintained as above. Animals had ad libitum access to food for 1 week. This was reduced to 10 h of food availability daily on week 2, with further reduction to 6 h daily by week 3. Finally, on week 4 and for the duration of the satiety studies, food availability was strictly maintained at 3 h daily (10.00–13.00 h). Animals (*n* = 8) received an oral dose of saline (0.9% NaCl) alone, or in combination with PPPH (100 mg/kg bw) immediately prior to regular food access at 10.00 h. Food intake was measured at 30 min intervals up to 180 min.

### Statistical analysis

Results were analysed using GraphPad PRISM 5.0 (San Diego, CA, USA), with data presented as mean ± SEM. Comparative analyses between groups were carried out using Student’s unpaired *t* test, one-way ANOVA with a Bonferroni post hoc test, or a two-way repeated measures ANOVA with a Bonferroni post hoc test where appropriate. Results were deemed significant once *p* < 0.05.

## Results

### Insulin secretion and cell viability following PPPH co-incubation with BRIN-BD11 cells

**Fig. 1:**
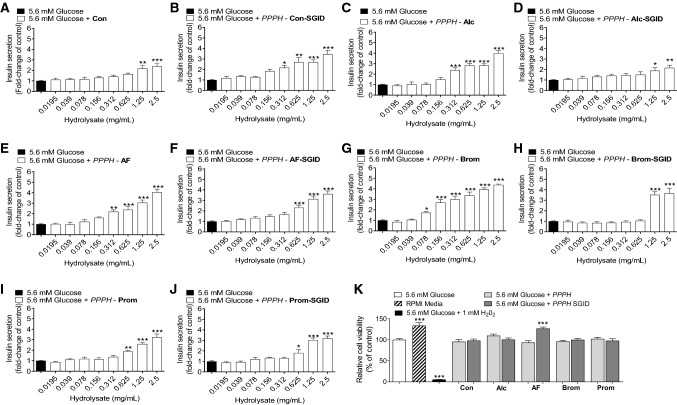
**a**–**k** Effects of PPPH (0.0195–2.5 mg/ml) on insulin release from clonal pancreatic BRIN-BD11 beta-cells at basal, 5.6 mM, glucose concentration. **k** Additionally, effects of a fixed (2.5 mg/ml) concentration of various protein hydrolysate on cell viability were also investigated. Values are mean ± SEM (*n* = 8). *p < 0.05, **p < 0.01, ***p < 0.001 compared to control 5.6 mM glucose (**a**–**k**). Con: aqueous/alkaline control, AF: Alcalase/Flavourzyme, Alc: Alcalase, Brom: Bromelain, Prom: Promod

Insulin secretion was determined over a 20 min co-incubation with PPPH supplemented glucose. Baseline insulin secretion was established utilising KRBB buffer supplemented with basal 5.6 mM or elevated 16.7 mM glucose. Several PPPH’s, subjected to different hydrolysis conditions, were employed along with SGID equivalents over an identical concentration range (0.0195–2.5 mg/ml). The aqueous/alkaline protein isolate which was subjected to similar hydrolysis condition to that of PPPH’s, albeit without the addition of enzyme, was employed as a control and elicited elevated (*p* < 0.01—*p* < 0.001) insulin secretion at 1.25 and 2.5 mg/ml at basal glucose concentration (Fig. 1a). Interestingly, its SGID equivalent presented with surprisingly high insulinotropic activity (*p* < 0.01-*p* < 0.001 at > 0.312 mg/ml) when tested at 16.7 mM glucose concentration in BRIN-BD11 cells (Fig. 1b). While potentially anomalous, improved potency (*p* < 0.01-*p* < 0.001 at > 0.156 mg/ml) following SGID, highlights the importance of more complete hydrolysis to the insulinotropic effect.

The inverse was true for PPPH’s. Alcalase PPPH stimulated insulin secretion (*p* < 0.001) from 0.312 mg/ml and above (Fig. 1c). However, following SGID, the effect on insulinotropic potency was negatively impacted, with bioactivity (*p* < 0.05 to *p* < 0.01) observed from 1.25 mg/ml and above (Fig. 1d). Alteration of the hydrolysis medium for Alcalase/Flavourzyme PPPH improved potency, with augmented (*p* < 0.01 to *p* < 0.001) insulin secretion from 0.312 mg/ml and above (Fig. 1e). Post-SGID, Alcalase/Flavourzyme PPPH displayed improved potency, increasing (*p* < 0.001) insulin secretion at 0.625 mg/ml or above (Fig. 1f). Of other digestion conditions, the bromelain PPPH exhibited promising insulin secretory actions, with improvements (*p* < 0.05—*p* < 0.001) compared to baseline at 0.078 mg/ml or above (Fig. 1g). SGID negatively impacted efficacy; however, potency was still impressive, enhancing (*p* < 0.001) insulin secretion from 1.25 mg/ml or higher (Fig. 1h). Somewhat unexpectedly, the Promod PPPH (Fig. 1i) and its SGID equivalent (Fig. 1j) displayed bioactivity over an identical concentration range (0.625–2.5 mg/ml) with only the magnitude of the increased insulin secretion being slightly impacted following SGID.

**Fig. 2:**
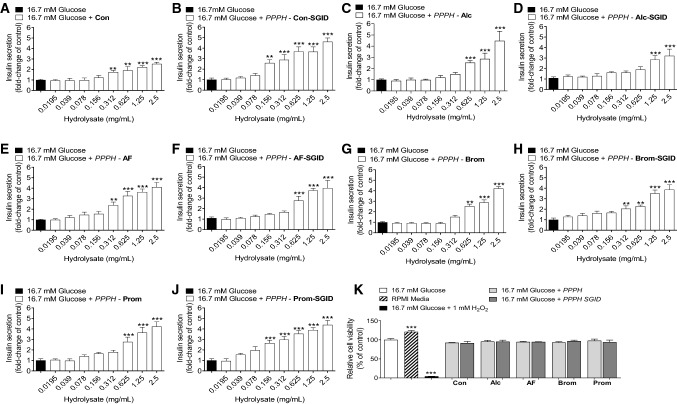
** a**–**k** Effects of PPPH (0.0195–2.5 mg/mL) on insulin release from clonal pancreatic BRIN-BD11 beta-cells at elevated, 16.7 mM, glucose concentration. **k** Additionally, effects of a fixed (2.5 mg/ml) concentration of various protein hydrolysate on cell viability were also investigated (K). Values are mean ± SEM (*n* = 8). **p < 0.01, ***p < 0.001 compared to control 16.7 mM glucose (**a**–**k**). Con: aqueous/alkaline control, AF: Alcalase/Flavourzyme, Alc: Alcalase, Brom: Bromelain, Prom: Promod

PPPH’s were further tested in the presence of 16.7 mM glucose. While potency was slightly altered, the magnitudes and trends involving SGID were largely the same for the aqueous, Alcalase, Alcalase/Flavourzyme PPPH’s and their SGID counterparts (Fig. 2a–f). The potency of both bromelain and Promod PPPH’s at elevated glucose was reduced, with respect to the 5.6 mM glucose data, but they retained a dose-dependent effect above 0.625 (Fig. 2g, i). SGID enhanced the potency of these PPPH’s in both cases (Fig. 2.H,J). Importantly, when tested at the highest concentration, cell viability was not negatively impacted by the inclusion of any PPPH at either 5.6 mM (Fig. 1k) or 16.7 mM glucose (Fig. 2k).

### Preservation of the incretin effect

As shown in Table 1, DPP-4 inhibition significantly increased following digestion with all proteolytic enzymes employed. Greatest inhibition was observed with PPPH’s generated with Alcalase/Flavourzyme or Alcalase alone, achieving DPP-4 IC_50_ values of 0.70 ± 0.02 and 0.94 ± 0.10 mg/ml, respectively. Bromelain and Promod PPPH’s had lower DPP-4 inhibitory activity with IC_50_ values of 1.34 ± 0.05 and 1.23 ± 0.05 mg/ml, respectively. IC_50_ values of the control and PPPH’s were significantly altered by SGID. The DPP-4 inhibitory activity mediated by the control and bromelain and Promod PPPH’s increased (*p* < 0.05) following SGID. In contrast, the DPP-4 inhibitory activity with Alcalase/Flavourzyme and Alcalase PPPH’s decreased (*p* < 0.05) following SGID. Thus, in the latter case peptides eliciting high DPP-4 inhibitory activity in the hydrolysate were degraded during SGID.

**Table 1:** DPP-4 inhibitory activity of PPPH’s and their SGID equivalents

Proteolytic activity	IC_50_ value (mg/ml)	
	PPPH	PPPH-SGID
Control (none)	1.91 ± 0.10^d^	1.09 ± 0.06^bc,^*
Alcalase + Flavourzyme	0.70 ± 0.02^a^	1.00 ± 0.03^bc,^*
Alcalase	0.94 ± 0.10^b^	1.14 ± 0.05^c,^*
Bromelain	1.34 ± 0.05^c^	0.95 ± 0.08^b,^*
Promod	1.23 ± 0.05^c^	0.78 ± 0.02^a,^*

Mean ± SD (*n*=3), IC_50_: inhibitory concentration that inhibits enzyme activity by 50%. *Indicates a significant difference (*p*< 0.05) in IC_50_ values following SGID

### Promotion of the incretin effect

**Fig. 3:**
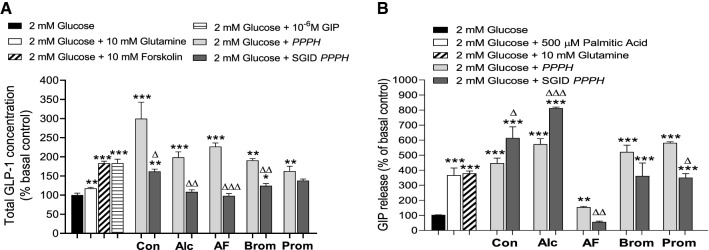
Effects of fixed concentration (2.5 mg/mL) of PPPH on the incretin effect through (a) GLP-1 release from GLUTag cells and (b) GIP release from STC-1 α-cells. Values are mean ± SEM (*n* = 4). *p* < 0.05, **p < 0.01, ***p < 0.001 compared to 2 mM glucose controls (**a**, **b**). ^Δ^*p* < 0.05, ^ΔΔ^*p* < 0.01, ^ΔΔΔ^*p* < 0.001 compared to the appropriate, non-SGID hydrolysate (**a**, **b**). Con: aqueous/alkaline control, AF: Alcalase/Flavourzyme, Alc: Alcalase, Brom: Bromelain, Prom: Promod

The effects of PPPH upon GLP-1 and GIP secretion was investigated via acute exposure of enteroendocrine GLUTag and STC-1 cell lines, respectively. Positive controls, glutamine (10 mM), forskolin (10 mM) and GIP (10^–6^ M) returned from 2- to fourfold (*p* < 0.05–*p* < 0.001) increases in GLP-1 secretion when compared to basal glucose (2 mM) control (Fig. 3a). Likewise, palmitic acid (500 μM) and glutamine (10 mM) showed a fourfold increase (*p* < 0.001) in GIP secretion compared to glucose control (Fig. 3b). PPPH’s (2.5 mg/ml) were subsequently co-incubated with 2 mM glucose to investigate their effects on hormone secretion.

The aqueous/alkaline protein control elicited a threefold increase (*p* < 0.001) in GLP-1 secretion compared to basal glucose control (Fig. 3a). Following SGID, bioactivity was reduced (*p* < 0.05); however, GLP-1 secretion remained elevated (*p* < 0.01) versus the 2 mM glucose control (Fig. 3a). Interestingly, the inverse was true for GIP secretion, where the protein control elicited a fourfold (*p* < 0.001) increase, but the SGID equivalent led to 5.8-fold (*p* < 0.001) increase in GIP secretion compared to glucose control (Fig. 3b). Alcalase PPPH displayed a twofold (*p* < 0.001) increase in GLP-1 secretion, but post-SGID it failed to raise secretion beyond the glucose control (Fig. 3a). For GIP secretion, Alcalase PPPH resulted in a sixfold (*p* < 0.001) increase which was improved post-SGID, with an eightfold (*p* < 0.001) upregulation (Fig. 3b). Little change was observed following addition of Flavourzyme, with the hydrolysate promoting a 2.2-fold increase in GLP-1 secretion (*p* < 0.001) accompanied by a loss of bioactivity following SGID (Fig. 3a). Unexpectedly, GIP secretion was impacted by Alcalase/Flavourzyme digestion, whereby the secretory activity was mildly elevated (1.2-fold; *p* < 0.01); however, following SGID, a significant reduction (*p* < 0.01) in secretion was observed (Fig. 3b). Bromelain PPPH produced twofold (*p* < 0.001) rise in GLP-1 secretion but only retained a 1.4-fold (*p* < 0.05) secretory response post-SGID (Fig. 3a). Bromelain PPPH and its SGID counterpart increased GIP secretion fivefold (*p* < 0.001) and 3.5-fold (*p* < 0.001), respectively, retaining relatively high bioactivity post-SGID (Fig. 3b). The same was largely true for Promod PPPH, displaying a 1.6-fold (*p* < 0.001) increase in GLP-1 secretion which was reduced to 1.4-fold (*p* < 0.05) following SGID (Fig. 3a). Again, GIP secretion was relatively well-retained with sixfold (*p* < 0.001) and 3.8-fold (*p* < 0.001) improvements in hormone secretion beyond control culture for Promod PPPH and its SGID equivalent, respectively (Fig. 3b).

### Mechanistic consequences of co-incubation with PPPH

**Fig. 4:**
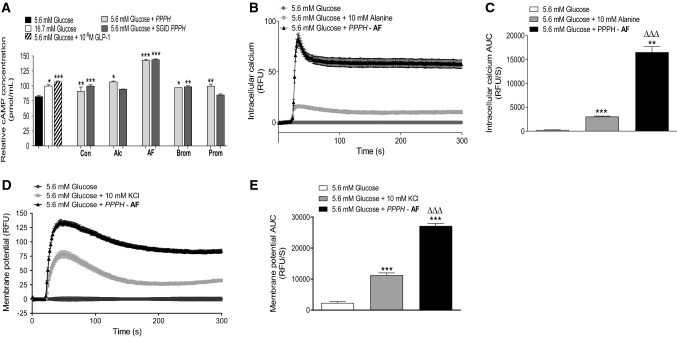
Mechanistic effects of co-incubation with fixed concentration (2.5 mg/ml) of PPPH following 20 min incubation with BRIN-BD11 cells. **a** PPPH’s and SGID equivalents, were tested for influence on cAMP concentration, **b** while only the Alcalase/Flavourzyme PPPH (AF) was employed for the study of intracellular calcium mobilisation and (**d**) membrane potential. Respective AUC values are also provided (**c**, **e**). Values are mean ± SEM (*n* = 3). *p < 0.05, **p < 0.01, ***p < 0.001 compared to 5.6 mM glucose control (**a**, **c**, **e**). ^ΔΔΔ^*p* < 0.001 compared to the 10 mM alanine positive control (**c**, **e**). Con: aqueous/alkaline control, AF: Alcalase/Flavourzyme, Alc: Alcalase, Brom: Bromelain, Prom: Promod

The cellular consequences following co-incubation of 2.5 mg/ml of PPPH were investigated in BRIN-BD11 cells supplemented with 5.6 mM glucose. Specifically, cyclic adenosine monophosphate (cAMP), intracellular calcium ([Ca^2+^]_i_) and membrane potential were tested under identical conditions. cAMP was initially used to screen PPPH’s. The positive controls, 16.7 mM glucose and GLP-1 (10^–6^ M), elevated (*p* < 0.05 and *p* < 0.001, respectively) intracellular cAMP versus 5.6 mM glucose control (Fig. 4a). All six non-SGID, PPPH’s raised cAMP production (*p* < 0.05–*p* < 0.001) in BRIN-BD11 cells (Fig. 4a), while only SGID, Alcalase and SGID, Promod PPPH’s failed to stimulate cAMP above the 5.6 mM control (Fig. 4a). Notably, Alcalase/Flavourzyme PPPH and its SGID counterpart, elicited the greatest rise in intracellular cAMP, upregulating 1.7-fold (*p* < 0.001) compared to 5.6 mM glucose control (Fig. 4a). As a result of constraints over assay availability, combined with the limited availability of SGID sample, only Alcalase/Flavourzyme PPPH was investigated further with respect to cellular signalling.

In terms of intracellular Ca^2+^ mobilisation, the positive control, alanine (10 mM), elicited a 15-fold increase (*p* < 0.001) in calcium mobilisation compared to the 5.6 mM glucose control culture (Fig. 4b, c). Alcalase/Flavourzyme PPPH greatly surpassed (*p* < 0.001) the positive control, increasing Ca^2+^ mobilisation 80-fold (*p* < 0.001) versus the 5.6 mM glucose control (Fig. 4b, c). With respect to membrane potential, potassium chloride (KCl, 30 mM) a potent membrane potentiating insulinotropic electrolyte, caused a 75-fold increase (*p* < 0.001) in membrane potential during acute co-incubation with 5.6 mM glucose (Fig. 4d, e). Further to increased Ca^2+^, Alcalase/Flavourzyme PPPH returned a 125-fold peak increase (*p* < 0.001) in membrane potential versus 5.6 mM glucose control in BRIN-BD11 cells (Fig. 4d, e).

### Effects of PPPH on in vitro glucose uptake

**Fig. 5:**
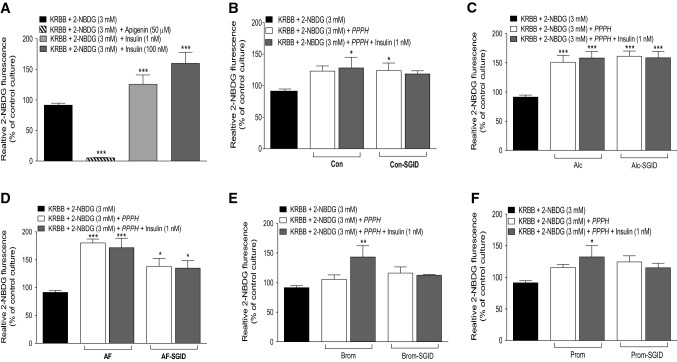
Effects of PPPH (2.5 mg/ml) on glucose uptake individually, or in combination with insulin (1 nM), in trans-differentiated 3T3-L1 adipocyte cells. Responsiveness of adipocyte cells was assessed via incubation with either apigenin (50 μfM), insulin (1 nM or 100 nM) (**a**). Adipocyte cells were further incubated with either Con/Con-SGID (**b**), Alc/Alc-SGID (**c**), AF/AF-SGID (**d**), Brom/Brom-SGID (**e**) or Prom/Prom-SGID (**f**) using fixed concentration of hydrolysate (2.5 mg/ml) for 1 h with 3 mM fluorescent glucose (2-NBDG). Values are mean ± SEM (*n* = 3). *p < 0.05, **p < 0.01, ***p < 0.001 compared to respective glucose control. Con: aqueous/alkaline control, AF: Alcalase/Flavourzyme, Alc: Alcalase, Brom: bromelain, Prom: Promod

The 3T3-L1 cell line was investigated following trans-differentiation from fibroblast to adipocyte cells. Apigenin control culture evoked a significant reduction (*p* < 0.001) of glucose uptake, via the inhibition of the GLUT-1 receptor, with the inverse for low (1 nM) and high (100 nM) insulin, causing 1.4- and 1.8-fold increases (*p* < 0.001) in glucose uptake, respectively (Fig. 5a). All PPPH’s and their SGID equivalents, were employed either alone at 2.5 mg/ml or in combination with basal insulin (1 nM).

Interestingly, while the protein control failed to show a significant increase in glucose on its own, in the presence of insulin a 1.5-fold increase (*p* < 0.05) was observed (Fig. 5b). The inverse was true following SGID causing significantly increased (*p* < 0.05) glucose uptake on its own, with surprising loss of effect in the presence of insulin (Fig. 5b). When co-incubated alone, Alcalase PPPH and its SGID equivalent both caused comparable, 1.5-fold (*p* < 0.001), increases in glucose uptake; however, when co-incubated with insulin, no additive effect was displayed (Fig. 5c). Alcalase/Flavourzyme PPPH, when incubated alone, caused a 1.8-fold increase (*p* < 0.001) in glucose uptake, with co-incubation with insulin not impacting greatly, demonstrating a 1.7-fold increase (*p* < 0.001) in uptake (Fig. 5d). Similarly, post-SGID, elicited a 1.38-fold (*p* < 0.05) rise in glucose uptake both with and without insulin present (Fig. 5d). Bromelain PPPH failed to improve glucose uptake when incubated on its own, with notable improvement presented as a 1.5-fold increase (*p* < 0.01) following insulin co-incubation (Fig. 5e). However, following SGID, the sample failed to produce an improvement in glucose uptake in the absence or presence of insulin (Fig. 5e). Similarly, Promod PPPH was only effective in the presence of insulin, when glucose uptake increased by 1.5-fold (*p* < 0.05), but this was lost following SGID, with the sample having no effect either alone or in the presence of insulin (Fig. 5f).

### Acute in vivo effects following oral administration of PPPH

**Fig. 6:**
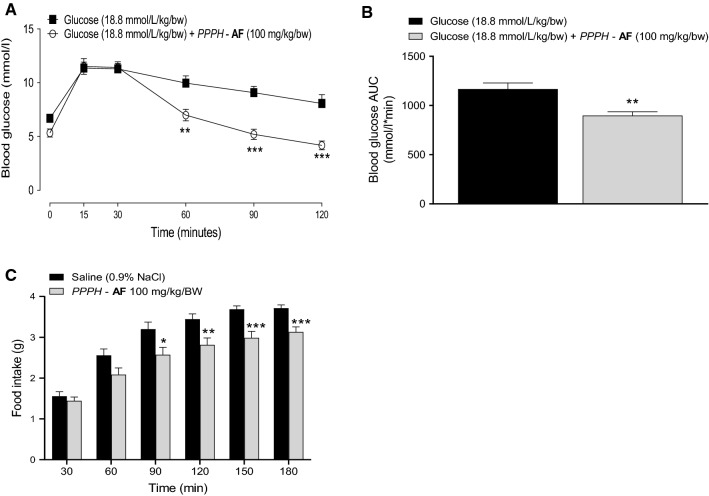
**a**, **b** Effects of a fixed dose (100 mg/kg/bw) of Alcalase/Flavourzyme PPPH (AF) on glucose tolerance in lean, overnight-fasted (16 h) NIH Swiss mice and (**c**) acute food intake in lean, diet-restricted HsD:Ola T0 mice. Animals (*n* = 8) received glucose (18.8 mmol/kg/bw) alone or in combination with hydrolysate (**a**) or saline (0.9% NaCl) alone or in combination with hydrolysate via oral gavage (**c**). Measurements were taken at regular intervals as indicated (**a**, **c**). Values are mean ± SEM (*n* = 8). *p < 0.05, **p < 0.01, ***p < 0.001 compared to glucose only (**a**, **b**) or saline only (**c**) controls

Following initial dose–response investigations (see Supplementary Figures), a 100 mg/kg/bw dose of Alcalase/Flavourzyme PPPH was employed for in vivo investigations. When co-administered with 18.8 mM glucose, PPPH significantly curbed rises in blood glucose at 60 min (*p* < 0.01), as well as 90 and 120 min (*p* < 0.001), when compared to glucose-only control (Fig. 6a). Additionally, the area under the curve (AUC) was significantly reduced (*p* < 0.01) compared to control (Fig. 6b). Positive glycaemic effects appear to be relatively short-lasting, with a delayed oral glucose challenge showing no anti-hyperglycaemic efficacy when delivered 4, 8 or 12 h post PPPH administration (data not shown).

Additionally, when co-administered with saline, PPPH (100 mg/kg) demonstrated significant reductions (*p* < 0.05 to *p* < 0.001) in food intake (16–20% from 90 to 180 min) when tested in diet-restricted animals (Fig. 6c).

## Discussion

While accepted that an increase in dietary protein intake can have positive effects on glycaemic control for T2DM patients [10]; expanding population sizes, coupled with pre-existing social inequality, continue to drive global protein malnutrition [45–47]. It is predicted that the global human population will reach 9 billion by 2050, meaning trends in population expansion are unlikely to be curbed. Thus, greater emphasis must be placed on ensuring food security [48]. Novel sources are being pursued to address this, one such being the exploration of marine sources like seaweeds, industry off-cuts and underutilised, or low value fish species, many of which are currently used as animal and farmed fish/shellfish feed [31, 49, 50]. Algal proteins are of particular interest due to a favourable amino acid composition, containing all nine essential amino acids [51], combined with a relatively high protein content, particularly for red seaweeds which often contain ≥ 30% of protein by dry weight [52].

It was recently established that chronic administration of an Alcalase/Flavourzyme PPPH can improve glycaemic control in STZ-induced diabetic mice [34]. Therefore, utilising an array of in vitro and acute in vivo techniques, the present study aimed to uncover causative mechanisms of PPPH-induced benefits in diabetes, investigating Alcalase/Flavourzyme PPPH while taking the opportunity to compare it to a number of other enzymatically produced PPPH’s.

Initial screening involved assessment of insulinotropic activity of PPPH’s in BRIN-BD11 cells, which have been previously utilised in the screening of enzymatically produced protein hydrolysates [25–27]. We hypothesised that activity would rely on the hydrolysis process, specifically the duration of hydrolysis and the protease used, ultimately leading to a different range of bioactive peptides within crude mixtures [53]. Our data appear to support this, particularly with respect to the choice of enzyme and the extent to which it hydrolyses *P. palmata* proteins and the MW of peptides therein. As reflected in the molecular distribution profiles (Supplementary Table 1), and accompanying GP-HPLC profiles (Supplementary Fig. 4), both Alcalase and Alcalase/Flavourzyme mediated a higher extent of hydrolysis of *P. palmata* proteins than bromelain and Promod. Also, all hydrolysates were further hydrolysed during the in vitro digestion process, which had either a positive or negative impact on bioactivity.

Insulinotropic effects of the protein control, were vastly improved following SGID, while the insulinotropic potency of Alcalase and Alcalase/Flavourzyme PPPH’s subjected to SGID was reduced rather than totally abolished, an encouraging finding. Intriguingly, particularly at 16.7 mM glucose, bromelain and Promod PPPH’s showed unaltered or even improved bioactivity post-SGID. Further analysis may uncover the possibility that glycation of specific components can affect their bioactivity, with the data suggesting that, for most PPPH’s, the potency of insulinotropic effect was greater at 16.7 mM than at 5.6 mM glucose. Given insulinotropic peptides, such as GIP, are demonstrated to have improved bioactivity following N-terminal glycation [54, 55], this may be plausible, but remains a hypothesis. Furthermore, the favourable MTT data showing no cellular cytotoxicity, demonstrated that the increased insulin release induced by PPPH was not a result of beta-cell lysis [56].

It is well established that both crude hydrolysates [32], and isolated peptide sequences [33], derived from *P. palmata,* have excellent DPP-4 inhibitory properties. As such, for the present set of PPPH’s, these findings have been corroborated. Furthermore, the effects were largely retained or even improved upon following SGID, indicating extensive oral bioavailability [25, 27]. While likely a less important factor in in vitro insulin secretion than in in vivo scenarios, previously identified presence of intra-islet DPP-4 [72], as well as expression within beta-cells themselves [73], means the influence of DPP-4 inhibitory effects to the insulinotropic effects of PPPH’s cannot be completely ruled out, but certainly warrant further investigation. The clinical success of DPP-4 inhibition is mediated through preservation of the incretin effect, and this mechanism is well established in management of T2DM [23, 57–59]. The present data demonstrates that: beyond preservation of the incretin effect, crude PPPH’s can directly influence incretin-mediated glycaemic improvements via the stimulation of GLP-1 and GIP secretion [60]. Thus, all PPPH’s enhanced both in vitro GLP-1 and GIP secretion in their unaltered states, a finding supported by previous findings of marine protein-hydrolysate-induced GLP-1 secretion [25, 27]. Intriguingly, the effects on GIP secretion were more resistant to SGID than those of GLP-1, with the effect at least retained for every hydrolysate except Alcalase/Flavourzyme PPPH. It is important to note that, given the crude nature of the hydrolysate mixtures, differing small MW peptides or free amino acid components may be responsible for each effect.

In vitro analysis of mechanistic consequences of PPPH co-incubation further highlights the ability of PPPH to influence insulin release. Membrane depolarisation, calcium mobilisation and generation of cAMP were employed to provide a general overview of activity. As such, cAMP upregulation suggests that small MW peptides may be stimulating secretion of hormones such as insulin, GLP-1 and GIP via G-coupled protein receptor activation [61]. These receptors are only activated by extracellular stimuli and can have potent cellular activation via promotion of internal signalling cascades [62, 63]. For example, activation of the GLP-1r via GLP-1 promotes a significant increase in cAMP production and glucose-dependent insulin secretion [63]. Indeed, each PPPH elicited cAMP elevation beyond baseline, with varying degrees of effect. In particular, Alcalase/Flavourzyme PPPH exhibited the most potent elevation, with full retention following SGID. Furthermore, this hydrolysate greatly influenced both membrane depolarisation and calcium mobilisation, beyond positive controls. While the direct effects on intracellular signalling appear to be multifaceted [62–64], further investigation is required to identify whether the bioactivity emanates from a potent singular peptide entity within the crude mixture, or if it arises from synergistic mechanisms of various peptides or amino acids [65].

3T3-L1 adipocyte cells were utilised to examine the ability of the PPPH and SGID samples to stimulate glucose uptake. These cells exhibit all the components of insulin receptor and signal transduction cascade and are frequently used to investigate insulin mediated glucose transport [66]. It is believed that compensatory upregulation of insulin production and secretion can result in impaired glucose transport into liver, skeletal muscle, and adipose tissue in T2DM [67]. Furthermore, it is postulated that dysfunctional peripheral glucose uptake, may contribute to insulin resistance in skeletal muscle [68]. The present data unveils a positive role for PPPH in glucose transport, with Alcalase and Alcalase/Flavourzyme PPPH’s increasing glucose uptake, independent of insulin, with the effect partially surviving SGID. Such findings may be related to the acute glucose tolerance test data. Thus, oral administration of Alcalase/Flavourzyme PPPH improved the oral glucose tolerance significantly from 60 min onwards of challenge initiation. While thought to be multifactorial in nature, improved glucose uptake could be a contributing factor [69]. Insulin determination was not possible during these experiments; however, previous findings have demonstrated that chronic administration of an equivalent dose of this hydrolysate (split into twice daily 50 mg/kg/bw dosage) improved non-fasting insulin levels, with accompanying improvements in circulating glucose and HbA_1c_, over the treatment period [34]. Additionally, we highlight a role for PPPH administration in satiety, with notable reduction from baseline evident from 90 to 180 min inclusive when tested in food deprived trained mice, again supported by previous findings [34].

Taken together, this dataset fortifies interest in PPPH with regards to management of T2DM. Knowledge of the previously established roles in DPP-4 inhibition [32] and partial reversal of STZ-induced diabetes [34] have been expanded upon. Here, we show clear roles for PPPH in direct insulin and incretin secretion, supported by cellular signalling data, as well as improvements in glucose utilisation and tolerance now identified. It is now accepted that particular combinations of individual amino acids, such as leucine, alanine and glutamine, at supraphysiological concentrations can augment insulin secretion [61]. We postulate that such a mechanism may be at play here with crude hydrolysate mixtures, thus future work may isolate, identify and characterise specific peptides, single amino acids or combinations thereof, small molecules or lipids from the most consistently promising of the hydrolysates, Alcalase/Flavourzyme PPPH [33]. The latter hydrolysate was particularly promising given its direct insulinotropic effects, accompanied by promising effects on the intracellular mechanisms linked to insulin release, along with positive effects on glucose uptake.

The study possesses some limitations, primarily that limited SGID sample availability did not permit inclusion of all test hydrolysates in our mechanistic investigations. In the present preliminary study, the findings observed herein on glycaemic control and insulin secretion could arise due to multiple different actions mediated by the PPPH peptides. These might involve for example, the inhibition of DPP-4 prolonging the bioactivity of GLP-1 and GIP incretin hormones or insulinotropic actions of free amino acids. However, extensive detailed mechanistic studies would be required to elucidate the various mechanisms, which is beyond the scope of the current study. Furthermore, a chronic study with isolated peptides would be warranted to increase our knowledge on the bioactivity residing in PPPH. Additionally, given all the hydrolysates assessed herein inhibit angiotensin converting enzyme (ACE) (data not shown) combined with our clearly identified effects on satiety, it may be interesting to investigate the influence PPPH can play on regulation of satiating hormones like cholecystokinin (CCK) and PYY(3–36), both of which are substrates for ACE [70, 71].

## Supplementary Information

Below is the link to the electronic supplementary material.

Supplementary file1 (PPTX 338 kb)
