# Supplementary material for: Macroalgal protein hydrolysates from Palmaria palmata influence the ‘incretin effect’ in vitro via DPP-4 inhibition and upregulation of insulin, GLP-1 and GIP secretion
Source: Eur J Nutr. 2021 Jun 3;60(8):4439–52. doi: 10.1007/s00394-021-02583-3 (PMC8572210; doi:10.1007/s00394-021-02583-3)
Supplement: Supplementary file 1 — Supplementary file1 (PPTX 338 kb) [file 394_2021_2583_MOESM1_ESM.pptx]

## Slide 1
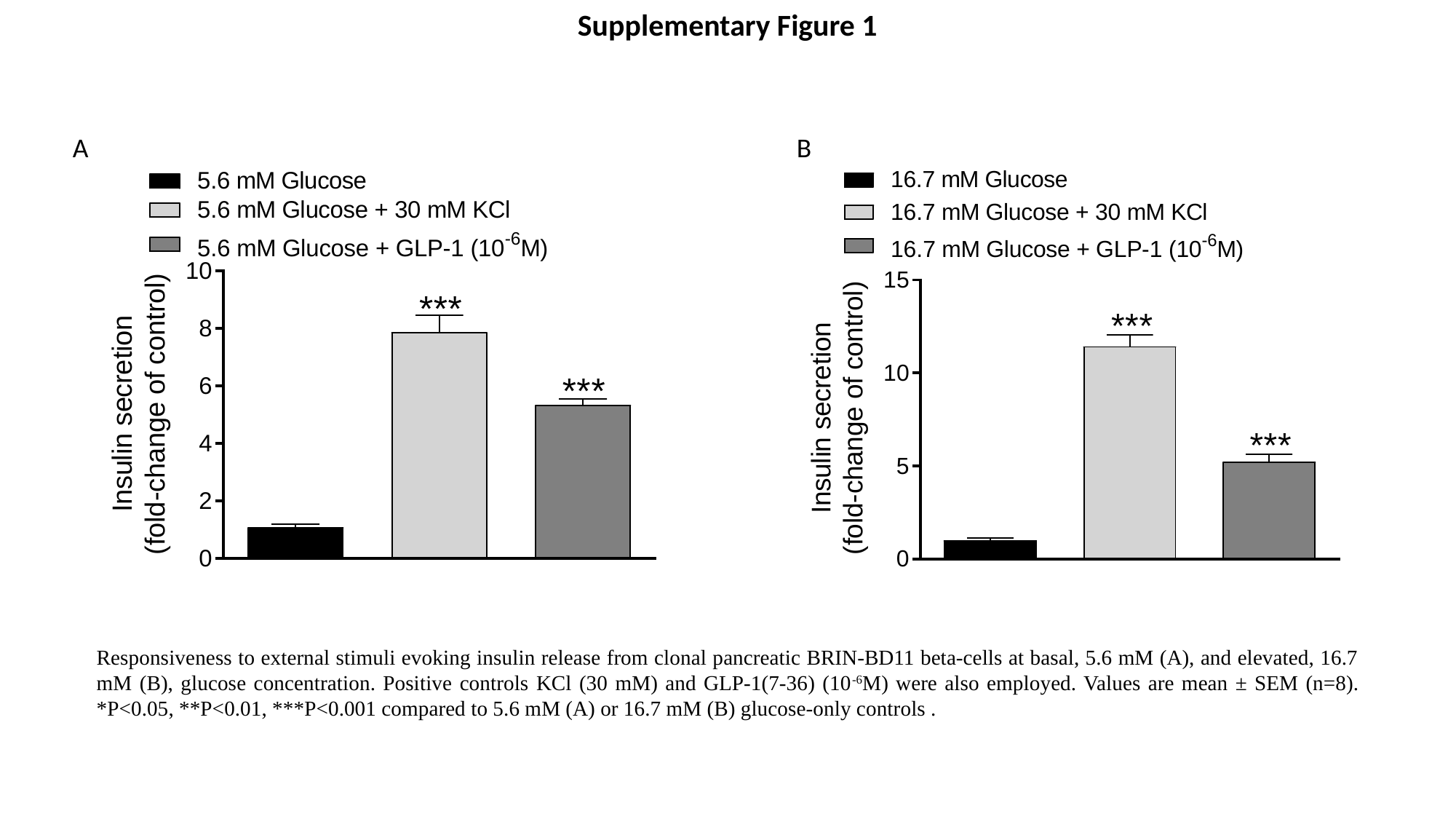

Supplementary Figure 1
A
B
Responsiveness to external stimuli evoking insulin release from clonal pancreatic BRIN-BD11 beta-cells at basal, 5.6 mM (A), and elevated, 16.7 mM (B), glucose concentration. Positive controls KCl (30 mM) and GLP-1(7-36) (10-6M) were also employed. Values are mean ± SEM (n=8). *P<0.05, **P<0.01, ***P<0.001 compared to 5.6 mM (A) or 16.7 mM (B) glucose-only controls .

## Slide 2
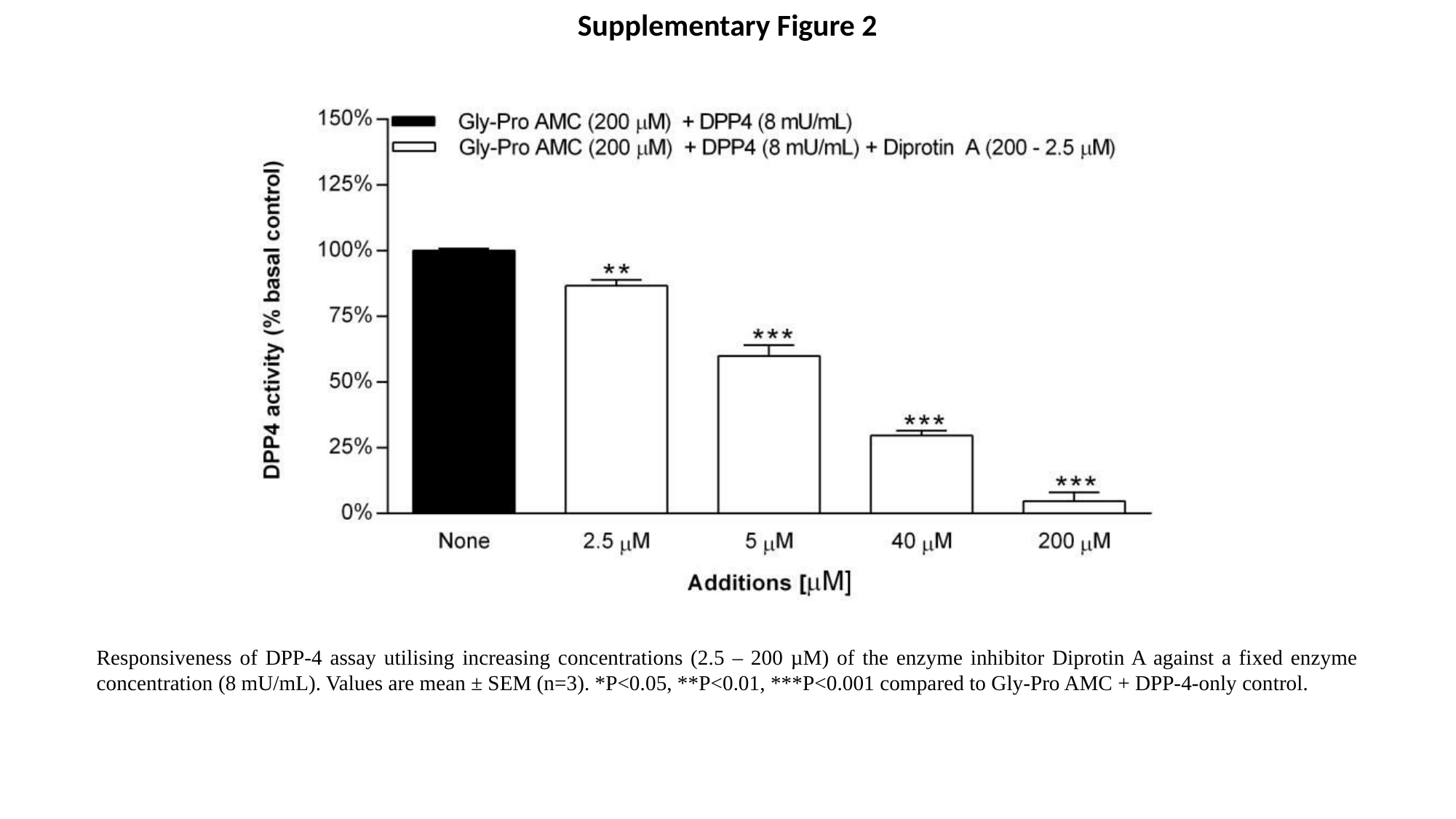

Supplementary Figure 2
Responsiveness of DPP-4 assay utilising increasing concentrations (2.5 – 200 µM) of the enzyme inhibitor Diprotin A against a fixed enzyme concentration (8 mU/mL). Values are mean ± SEM (n=3). *P<0.05, **P<0.01, ***P<0.001 compared to Gly-Pro AMC + DPP-4-only control.

## Slide 3
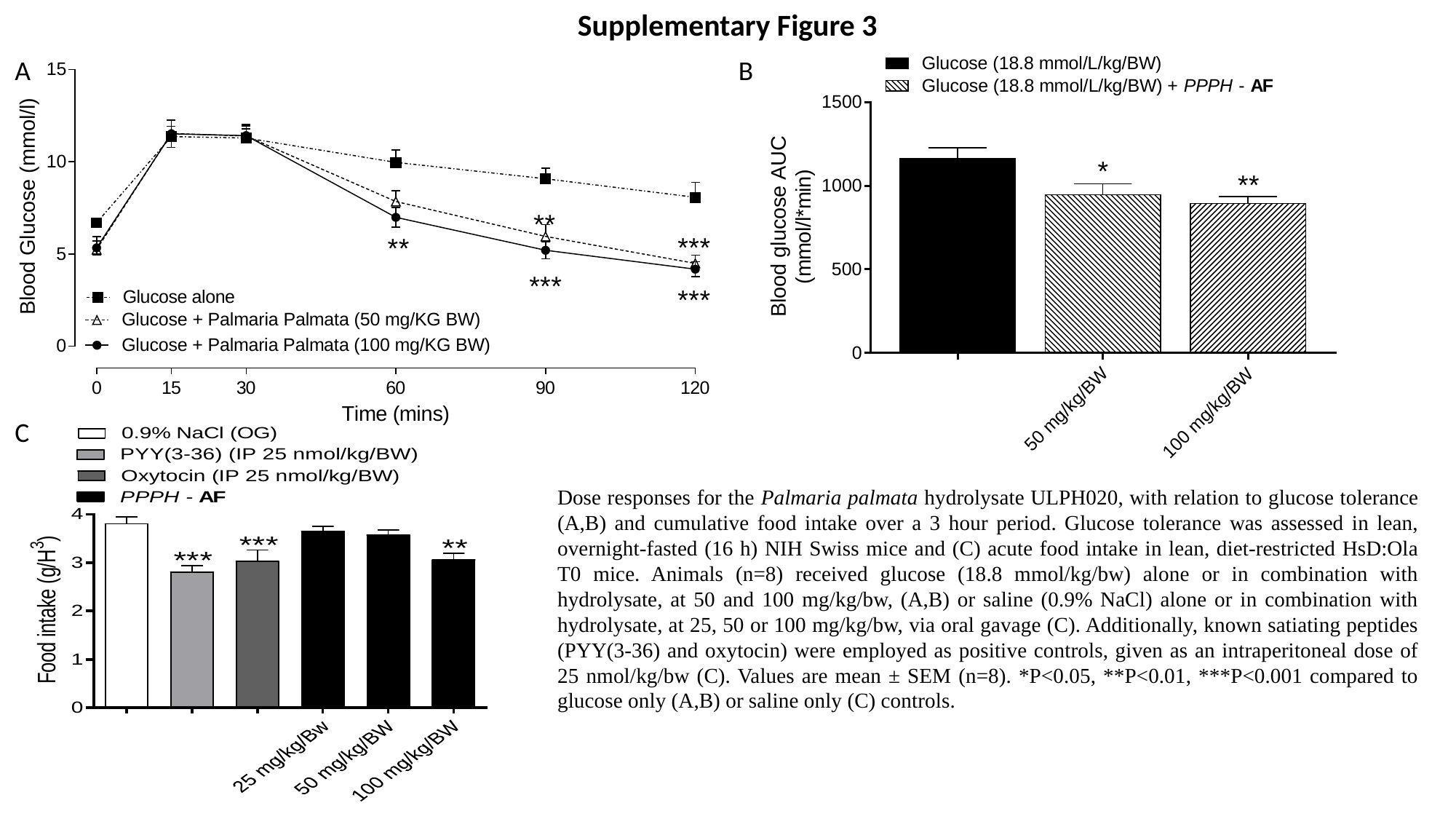

Supplementary Figure 3
A
B
C
Dose responses for the Palmaria palmata hydrolysate ULPH020, with relation to glucose tolerance (A,B) and cumulative food intake over a 3 hour period. Glucose tolerance was assessed in lean, overnight-fasted (16 h) NIH Swiss mice and (C) acute food intake in lean, diet-restricted HsD:Ola T0 mice. Animals (n=8) received glucose (18.8 mmol/kg/bw) alone or in combination with hydrolysate, at 50 and 100 mg/kg/bw, (A,B) or saline (0.9% NaCl) alone or in combination with hydrolysate, at 25, 50 or 100 mg/kg/bw, via oral gavage (C). Additionally, known satiating peptides (PYY(3-36) and oxytocin) were employed as positive controls, given as an intraperitoneal dose of 25 nmol/kg/bw (C). Values are mean ± SEM (n=8). *P<0.05, **P<0.01, ***P<0.001 compared to glucose only (A,B) or saline only (C) controls.

## Slide 4
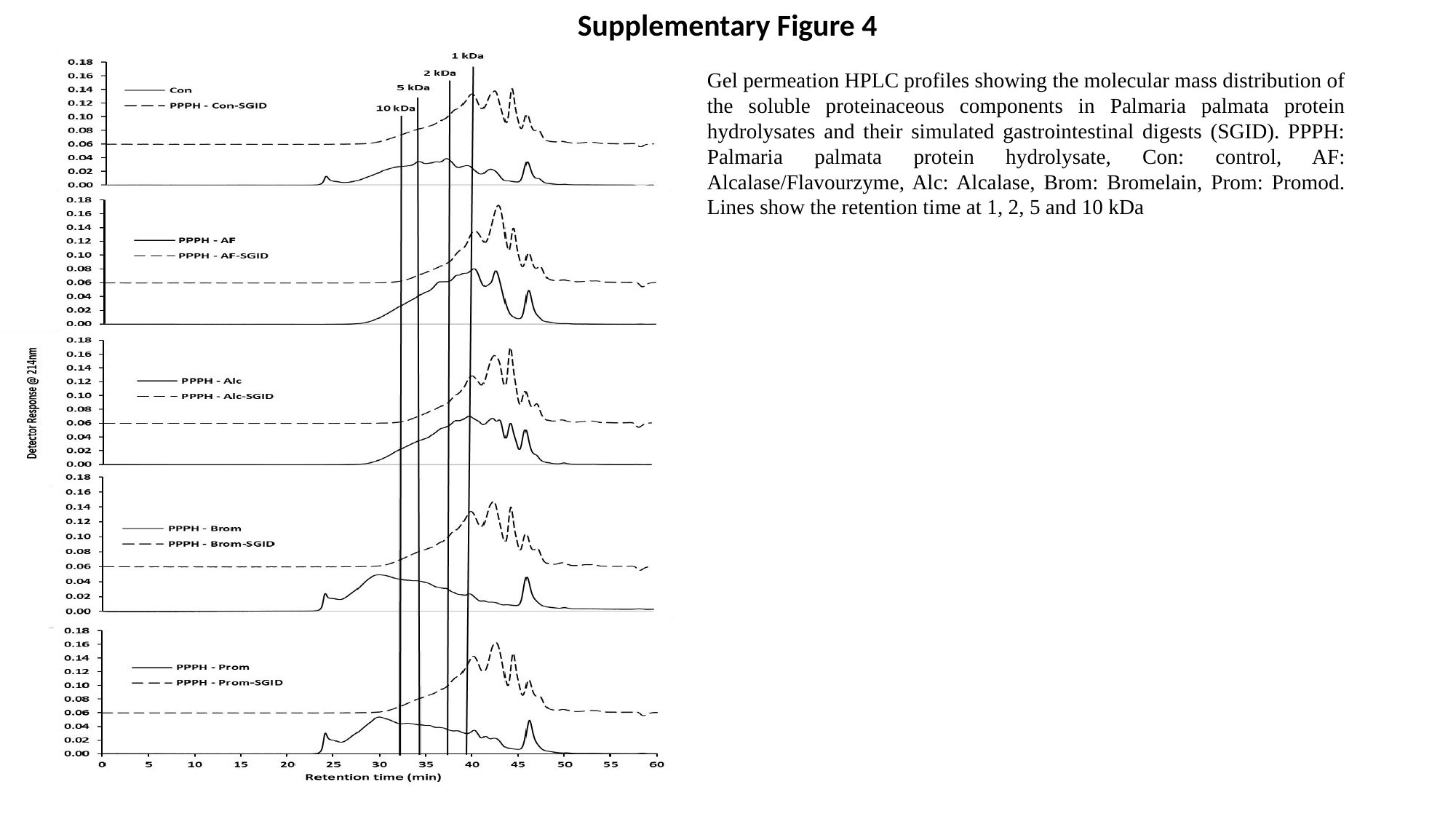

Supplementary Figure 4
Gel permeation HPLC profiles showing the molecular mass distribution of the soluble proteinaceous components in Palmaria palmata protein hydrolysates and their simulated gastrointestinal digests (SGID). PPPH: Palmaria palmata protein hydrolysate, Con: control, AF: Alcalase/Flavourzyme, Alc: Alcalase, Brom: Bromelain, Prom: Promod. Lines show the retention time at 1, 2, 5 and 10 kDa

## Slide 5
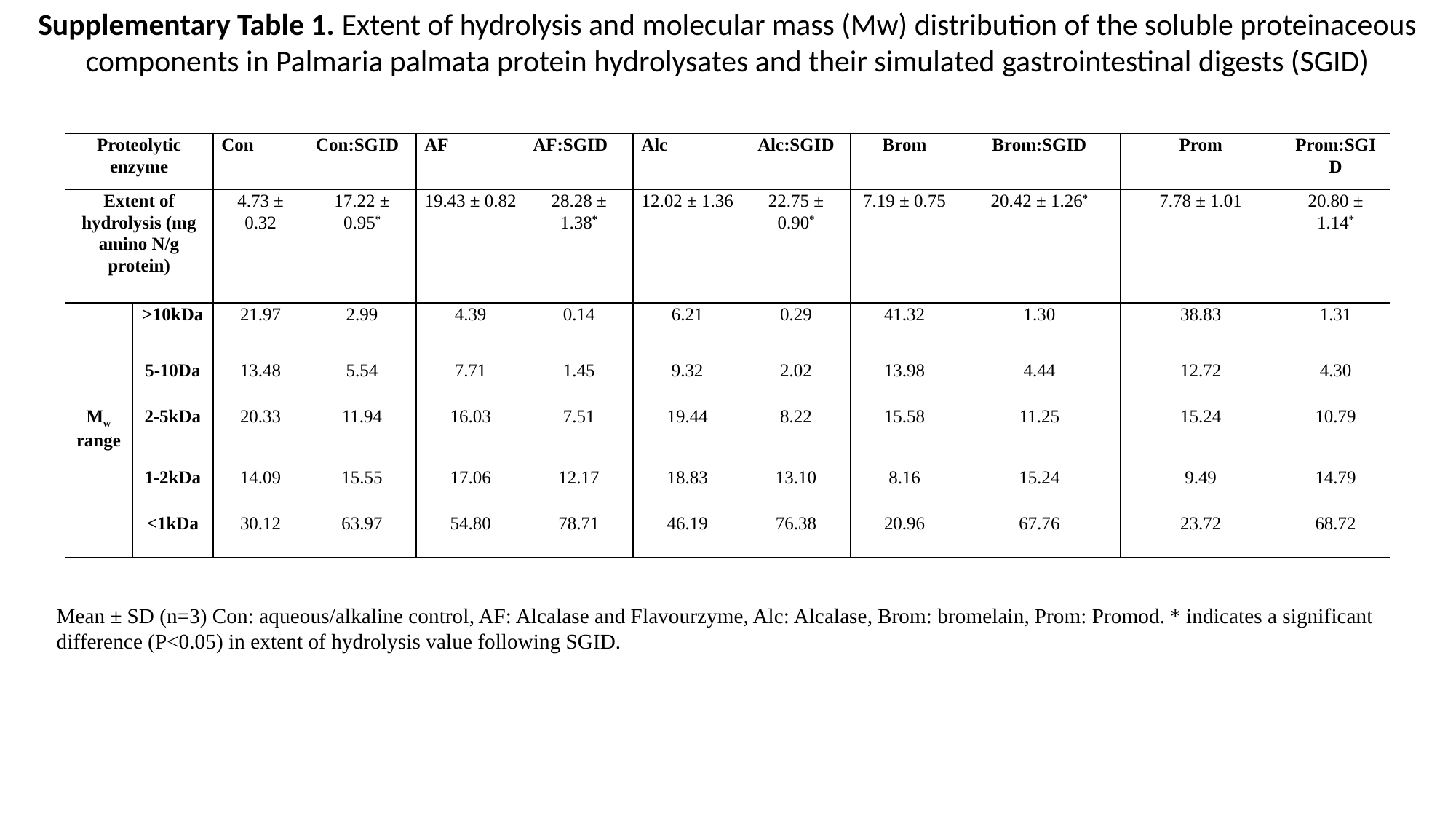

Supplementary Table 1. Extent of hydrolysis and molecular mass (Mw) distribution of the soluble proteinaceous components in Palmaria palmata protein hydrolysates and their simulated gastrointestinal digests (SGID)
| Proteolytic enzyme | | Con | Con:SGID | AF | AF:SGID | Alc | Alc:SGID | Brom | Brom:SGID | Prom | Prom:SGID |
| --- | --- | --- | --- | --- | --- | --- | --- | --- | --- | --- | --- |
| Extent of hydrolysis (mg amino N/g protein) | | 4.73 ± 0.32 | 17.22 ± 0.95\* | 19.43 ± 0.82 | 28.28 ± 1.38\* | 12.02 ± 1.36 | 22.75 ± 0.90\* | 7.19 ± 0.75 | 20.42 ± 1.26\* | 7.78 ± 1.01 | 20.80 ± 1.14\* |
| | >10kDa | 21.97 | 2.99 | 4.39 | 0.14 | 6.21 | 0.29 | 41.32 | 1.30 | 38.83 | 1.31 |
| | 5-10Da | 13.48 | 5.54 | 7.71 | 1.45 | 9.32 | 2.02 | 13.98 | 4.44 | 12.72 | 4.30 |
| Mw range | 2-5kDa | 20.33 | 11.94 | 16.03 | 7.51 | 19.44 | 8.22 | 15.58 | 11.25 | 15.24 | 10.79 |
| | 1-2kDa | 14.09 | 15.55 | 17.06 | 12.17 | 18.83 | 13.10 | 8.16 | 15.24 | 9.49 | 14.79 |
| | <1kDa | 30.12 | 63.97 | 54.80 | 78.71 | 46.19 | 76.38 | 20.96 | 67.76 | 23.72 | 68.72 |
Mean ± SD (n=3) Con: aqueous/alkaline control, AF: Alcalase and Flavourzyme, Alc: Alcalase, Brom: bromelain, Prom: Promod. * indicates a significant difference (P<0.05) in extent of hydrolysis value following SGID.
